# VFQB: A Novel Deep Learning Model for Rolling Bearing Fault Diagnosis

**DOI:** 10.3390/s25092678

**Published:** 2025-04-24

**Authors:** Zhiru Xiao, Yanfang Xu, Junjie Cui

**Affiliations:** 1 College of Mechatronic Engineering, North University of China, Taiyuan 030051, China; s202201043@st.nuc.edu.cn (Z.X.); 20020008@nuc.edu.cn (J.C.); 2 Shanxi Key Laboratory of High-End Equipment Reliability Technology, North University of China, Taiyuan 030051, China

**Keywords:** fault diagnosis, variational mode decomposition, fast Fourier transform, bidirectional gated recurrent unit, quadratic neural network

## Abstract

In rolling bearing fault diagnosis, weak features are often masked by complex environmental conditions, blurring the original fault signals and reducing diagnostic accuracy. To address this issue, we propose the VMD/FFT-Quadratic-BiGRU diagnostic model. First, the original vibration signals are processed with variational mode decomposition (VMD) and fast Fourier transform (FFT) and then stacked as quadratic neural network inputs. Next, a Bidirectional Gated Recurrent Unit (BiGRU) module is introduced to capture the temporal characteristics of the feature signals. An attention mechanism is then applied to assign weights to the hidden layers of the BiGRU network. Finally, fault diagnosis is performed using a fully connected layer and softmax classifier. Experimental results demonstrate that this model significantly enhances the ability to capture weak fault features in complex environments. The fault diagnosis accuracy reaches 100% on both datasets, showing improvements of 2.68% and 1.58% over models without the quadratic network. Additionally, comparisons with other models in noisy environments show that the proposed model exhibits superior noise suppression capabilities, further highlighting its robustness and diagnostic accuracy.

## 1. Introduction

With the rapid advancement of industrial automation and intelligent manufacturing, fault diagnosis technology has become an indispensable component in enhancing the stability of mechanical systems, optimizing production processes, and effectively reducing maintenance costs [1,2,3]. Rolling bearings, as critical rotating components in mechanical equipment, are susceptible to wear, fatigue, cracking, and other forms of failure during prolonged operation in high-load, complex environments. These failures often result in increased vibration, performance degradation, and, in severe cases, equipment shutdown [4,5,6]. Timely and accurate diagnosis of bearing faults is crucial for improving equipment reliability, minimizing economic losses, and ensuring production safety [7,8]. However, bearing fault signals are typically influenced by significant noise and non-stationary characteristics, posing substantial challenges to the extraction and classification of fault features. Consequently, numerous advanced methods have been proposed by researchers to address these challenges.

Traditional bearing fault diagnosis methods primarily rely on statistical analysis, expertise, and signal processing techniques such as the Fourier transform and wavelet transform, assessing the bearing’s operational status by extracting key features from vibration signals [9,10,11]. Among these techniques, wavelet transform has been widely used in early signal processing and fault diagnosis, and certain results have been achieved [12,13]. However, in modern complex industrial environments, wavelet transform faces many challenges when dealing with signals containing multiple noises, and its ability to identify fault features is often limited [14,15]. The Fourier transform analyzes frequency components by decomposing a signal into a set of sinusoids, which is suitable for spectral analysis of smooth signals but is ineffective in dealing with non-smooth signals [16,17]. In contrast, VMD, which is based on the local information of the signal for adaptive decomposition, is able to effectively separate the different components and accurately capture the frequency characteristics of non-smooth signals over time, even in the presence of non-smooth signals [18]. However, despite the partial success of these methods, their performance relies on a large amount of expert knowledge and still exhibits limitations in extracting effective features [9].

With the rapid development of artificial intelligence and big data technologies, data-driven fault diagnosis methods have garnered significant attention [19,20]. For instance, Hu et al. [21] proposed a multi-scale, multi-frequency branching interactive spatio-temporal sequence prediction network for predicting the remaining service life of railroad electromechanical equipment. Li et al. [22] introduced a joint-attention feature transfer network to address the category imbalance issue in industrial data. Kamil et al. [23] proposed the BiGRU-CNN model for real-time monitoring and technical status diagnosis of small unmanned aerial vehicle units. Niu et al. [24] leveraged CNN layers and a BiGRU to extract high-dimensional features and temporal dependencies from historical sequences, demonstrating strong performance compared to other models. Li et al. [14] combined graph convolutional networks with a residual module to enhance the model’s ability to capture localized features in signals. Zhao et al. [25] integrated the unsupervised feature learning capabilities of auto encoders with the powerful feature extraction abilities of CNN for fault detection and classification. Li et al. [26] proposed an ACWOS fault diagnosis method based on clustering weighted oversampling to solve the problem of bearing fault diagnosis when the operating conditions change and the data are unbalanced.

In recent years, research by both domestic and international scholars in the field of fault diagnosis has focused on innovative applications such as generative adversarial networks (GANs), lightweight model design, digital twin technology, etc. Pham et al. [27] proposed an improved GANs-based fault diagnosis method for rolling bearings, which solves the misclassification problem of the traditional CNN model in the insufficient-data scenario by generating two-dimensional time-frequency representation data of the acoustic emission signals. The method performs well in low-speed and composite fault datasets; however, the study does not consider the computational efficiency limitations in real-time diagnosis scenarios. Li et al. [28] constructed a multiscale fault evolution digital twin model for the entire life cycle of rolling bearings and achieved accurate prediction of the fault expansion mechanism through dynamic excitation mapping and real-time data updating. Li et al. [29] developed a frequency-time multimodal Transformer model, which fuses frequency-domain feature maps and time-domain feature vectors through multivariate decomposition and discrete wavelet transform. However, the high complexity of this model makes it difficult to deploy in resource-constrained edge devices. Zhong et al. [30] designed a simplified fast GANs and triple migration learning framework, which significantly reduces the time of GANs data generation. The time required for GANs to generate data was significantly reduced, and the model’s generalization ability was improved by joint training with open-source data, synthetic data, and real data. Niu et al. [31] proposed a fault diagnosis method based on a rolling element separation signal processing technique combined with a lightweight convolutional network. This approach reduces computational cost through channel sharing and unidirectional spatial convolution, and demonstrates strong robustness in noisy environments. However, its mechanical structure feature extraction process relies on manual design and does not achieve end-to-end adaptive optimization.

Despite the progress made by these innovative approaches, GANs are prone to problems such as gradient vanishing, pattern crashing, and oscillations during the training process, resulting in unreliable quality of the generated fault data. While lightweight models reduce resource consumption by reducing the number of parameters and computational complexity, they often sacrifice the expressive power of the model, which in turn affects diagnostic accuracy. Digital twin technology imposes high performance requirements when processing time-series data; thus, accurately capturing early signs of faults in complex environments remains a key challenge in current research.

These methods improve the ability to identify fault signatures in complex environments through finer signal analysis and processing. However, accurately capturing early fault signs under high noise and low signal-to-noise ratio conditions remains one of the major challenges in current research [32].

In this paper, a VMD/FFT-Quadratic-BiGRU model is proposed, aiming at the combined use of signal decomposition, feature enhancement, and temporal modeling capabilities to achieve effective extraction and fusion of weak features, along with improved noise immunity. The main innovations of the model are primarily reflected in the following aspects:

(1) Improved feature extraction method: A parallel processing strategy combining VMD and FFT is employed to process bearing vibration signals, enabling the extraction of both time-domain and frequency-domain feature sets of the bearing.
(2) Comparison with existing methods: We introduce the structure of combining a quadratic network and BiGRU and construct a diagnostic model with stronger noise robustness. The quadratic network enhances the feature signals through nonlinearities to effectively suppress the influence of noise; the BiGRU further refines the time series features through bi-directional time-dependent modeling to ensure the accuracy and robustness of fault classification.

The content of this paper is as follows:

(1) A model is proposed for bearing fault diagnosis, and a quadratic network is introduced to enhance the feature extraction capability.
(2) VMD and FFT are combined for signal preprocessing to effectively extract time–frequency domain information, while the BiGRU is used to capture time series features and improve the accuracy of fault classification.
(3) The effectiveness of the proposed model is verified by the publicly available CWRU dataset and several comparative experiments.

The remainder of the manuscript is organized as follows. In Section 2, related work is introduced and the working principle of the proposed method is described. Section 3 presents the experimental study, demonstrating the application and comparison of the proposed method across different datasets. Section 4 provides the conclusion, which summarizes the key findings of the paper.

## 2. Basic Principles and Model Structure

### 2.1. Principle of Variational Mode Decomposition

VMD is an adaptive signal decomposition technique that utilizes a non-recursive approach to decompose a complex signal into a series of intrinsic mode functions (IMFs) with distinct frequency characteristics and amplitude variations. This decomposition is performed according to a predetermined modal number *K* and penalty factor *α* [33]. VMD overcomes the uncertainty associated with the number of IMFs in traditional Empirical Mode Decomposition (EMD) methods, as well as the issues of endpoint effects and mode mixing. Consequently, it more effectively highlights the characteristic information of the signal [34]. The VMD represents the decomposition problem as a solution problem with a constrained model, and its constrained variational expression can be expressed as [33]:

(1) $$\left\{\begin{array}{l} \underset{\left\{u_{k}\right\},\left\{\omega_{k}\right\}}{min}\left\{\sum_{k}{\left\|\partial_{t}\left[\left(\delta \left(t\right)+\frac{j}{\pi t}\right)*u_{k}\left(t\right)\right]e^{-j\omega_{k}t}\right\|}_{2}^{2}\right\}\\ s.t.\;\sum_{k}u_{k}\left(t\right)=f\left(t\right) \end{array}\right.$$

where $f\left(t\right)$ is the input signal, $\;u_{k}\left(t\right)$ is the decomposed mode, $\partial_{t}$ is the partial derivative of the function over time, and $\delta \left(t\right)$ is the unit pulse function.

By introducing the quadratic penalty factor and the augmented Lagrangian function, the constrained variational problem is transformed into an unconstrained variational problem, and the following results are obtained:

(2) $$\begin{array}{l} L\left(\left\{u_{k}\right\},\left\{\omega_{k}\right\},\lambda \right)=\alpha {\sum_{k}\left\|\partial t\left[\left(\delta \left(t\right)+\frac{j}{\pi t}\right)*u_{k}\left(t\right)\right]e^{-j\omega_{k}t}\right\|}_{2}^{2}\\ +{\left\|f\left(t\right)-\sum_{k}u_{k}\left(t\right)\right\|}_{2}^{2}+\langle \lambda \left(t\right),f\left(t\right)-\sum_{k}u_{k}\left(t\right)\rangle \end{array}$$

where *α* is the quadratic penalty factor, *λ* is the Lagrange operator, and 〈,〉 represents the inner product.

The minimum value of Equation (3) is found by alternately updating each component and the center frequency using the alternating direction multiplier method:

(3) $${\hat{u}}_{k}^{n+1}\left(\omega \right)=\frac{f\left(\omega \right)-\sum_{i<k}{\hat{u}}_{i}^{n+1}\left(\omega \right)-\sum_{i>k}{\hat{u}}_{i}^{n+1}\left(\omega \right)+\frac{{\hat{\lambda }}^{n}\left(\omega \right)}{2}}{1+2\alpha {\left(\omega -\omega_{k}^{n}\right)}^{2}}$$

(4) $$\omega_{k}^{n+1}=\frac{\int_{0}^{\infty }\omega {\left|{\hat{u}}_{k}^{n+1}\left(\omega \right)\right|}^{2}d\omega }{\int_{0}^{\infty }{\left|{\hat{u}}_{k}^{n+1}\left(\omega \right)\right|}^{2}d\omega }$$

(5) $${\hat{\lambda }}^{n+1}(\omega )={\hat{\lambda }}^{n}\left(\omega \right)+\tau \left[\hat{f}\left(\omega \right)-\sum_{k}{\hat{u}}_{k}^{n+1}\left(\omega \right)\right]$$

### 2.2. Principle of the Bidirectional Gated Recurrent Unit

**Figure 1:**
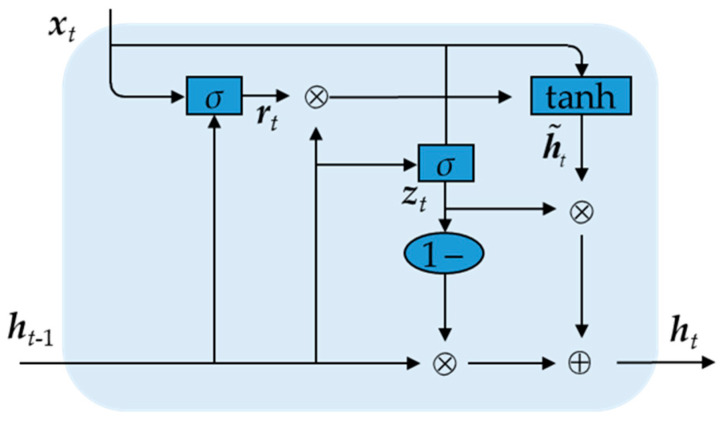
Basic structure of GRU.

The basic structure of the GRU is shown in Figure 1. The GRU has an update gate $z_{t}$ and a reset gate $r_{t}$. The update gate determines how much of the current hidden state of the time step should be inherited from the previous moment; the reset gate decides how much the current input affects the current hidden state [35]. Through these casting mechanisms, the GRU can better capture long-term dependence and avoid gradient loss or gradient explosion [36,37].

When input $x_{t}$ at moment *t* and the output $h_{t-1}$ at the previous moment are used as inputs,

(6) $$r_{t}=\sigma \left(W_{r}x_{t}+U_{r}h_{t-1}+b_{r}\right)$$

(7) $$z_{t}=\sigma \left(W_{z}x_{t}+U_{z}h_{t-1}+b_{z}\right)$$

(8) $${\tilde{h}}_{t}=\tan h\left(W_{h}x_{t}+U_{h}\left(r_{t}⊙h_{t-1}\right)+b_{h}\right)$$

(9) $$h_{t}=z_{t}⊙{\tilde{h}}_{t}+\left(1-z_{t}\right)⊙h_{t-1}$$

where ${\tilde{h}}_{t}$ denotes the candidate hidden layer state; $h_{t-1}$ and $h_{t}$ represent the hidden layer state at moments t−1 and *t*, respectively; $W_{r}$, $W_{z}$, $W_{h}$, $U_{r}$, $U_{z}$, $U_{h}$ are weight matrices; $b_{r}$, $b_{z}$, $b_{h}$ denote the bias vectors; × denotes the scalar multiplication of the matrix and + denotes the addition of the matrix; *σ* denotes the sigmoid activation function and tanh denotes the hyperbolic tangent activation function; and ⊙ indicates point multiplication.

**Figure 2:**
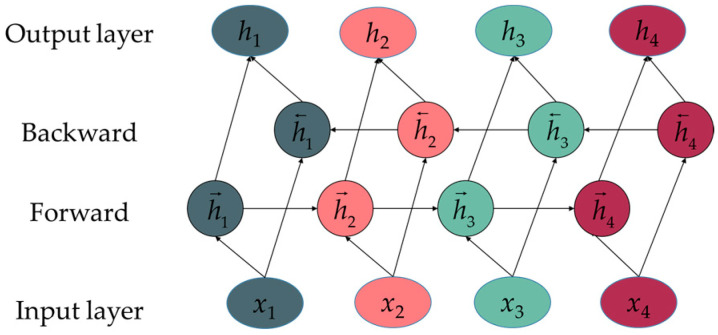
BiGRU structure.

The basic structure of the BiGRU is shown in Figure 2. The BiGRU consists of a forward GRU network and a reverse GRU network, which are responsible for capturing historical and future information, respectively. By integrating the outputs of these two networks based on their respective time positions, the model enhances memory capacity and prediction accuracy [38].

### 2.3. Principle of Quadratic Neural Network

The artificial neuron model views neurons as linear functions of input vectors to produce outputs, a function of the model, as in Equation (10) [39]:

(10) $$f\left(x\right)=\sum_{i=0}^{n}w_{i}x_{i}$$

where $w_{i}$ denotes the weights, $x_{i}$ denotes the inputs, and then $f\left(x\right)$ is nonlinearly processed, e.g., by a sigmoid function, as in Equation (11).

(11) $$y=\sigma \left(f\left(x\right)\right)$$

where *y* denotes the neuron output.

Fan et al. proposed a quadratic neural network [40], which replaces the traditional neurons with quadratic neurons consisting of inner products and power terms of the input vectors. Given an input sample $x\in {\mathbb{R}}^{1\times n}$, $x=\left[x_{1},x_{2},\cdots ,x_{n}\right]$, the quadratic convolution operation can be expressed as:

(12) $$Q(x)=\sigma \left(\left(x*w^{r}+b^{r}\right)⊙\left(x*w^{g}+b^{g}\right)+(x⊙x)*w^{b}+c\right)$$

where ∗ denotes the convolution operation, ⊙ denotes the Hadamard product, $w^{r}\in {\mathbb{R}}^{k\times 1}$, $w^{g}\in {\mathbb{R}}^{k\times 1}$, and $w^{b}\in {\mathbb{R}}^{k\times 1}$ denote the three different convolution kernels for the weights, *σ*(⋅) is the activation function (e.g., ReLU), and $b^{r}$, $b^{g}$, *c* denote the bias corresponding to these convolution kernels.

While traditional neurons require an exponential number of neurons [40], quadratic neurons require only a polynomial number of neurons, while exhibiting superior performance in approximating radial basis functions, which gives quadratic networks a higher capability in feature extraction [41,42]. In addition, quadratic networks are capable of polynomial approximation, whereas traditional neural networks can only achieve segmental approximation through nonlinear activation functions. Since the distribution of real data is usually nonlinear, these properties help to enhance the generalization and representation ability of neural networks.

### 2.4. VMD/FFT-Quadratic-BiGRU Model Construction

**Figure 3:**
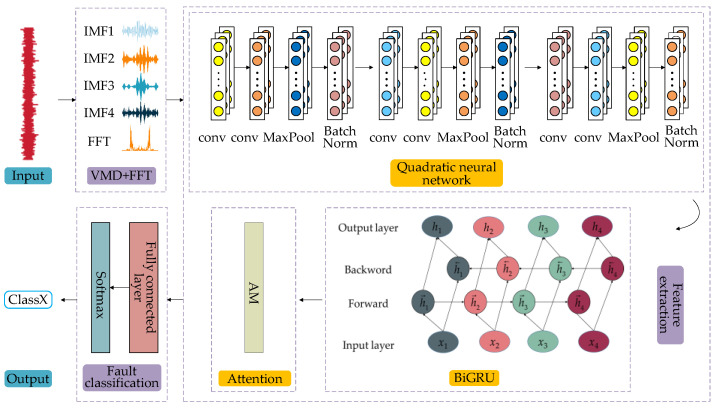
VFQB fault diagnosis model.

In this paper, a bearing fault diagnosis model VMD/FFT-Quadratic-BiGRU (VFQB) is proposed, as shown in Figure 3, which consists of three main parts: preprocessing, feature extraction, and fault classification.

In the preprocessing stage, the signal is processed using a combination of VMD and FFT to extract time-domain and frequency-domain features, respectively. This dual approach allows for the simultaneous capture of both global frequency-domain information and local time-domain characteristics of the signal. Additionally, the decomposition feature of VMD effectively isolates signals across different frequency bands, reducing noise interference, while the frequency-domain features derived from FFT remain stable even in the presence of strong noise. The fusion of these two feature sets enhances the robustness of the input data.

In the feature extraction phase, the model incorporates a quadratic network layer and a BiGRU to comprehensively extract features and capture the dynamic information of the signal. The quadratic network layer consists of a convolutional layer that captures the local spatial characteristics of the input data, followed by a maximum pooling layer that reduces the number of parameters and computational complexity through dimensionality reduction, while preserving essential information. These operations enable the model to extract local features of the signal and significantly enhance feature representation. The BiGRU module captures both forward and backward information from the data sequences, facilitating the exploration of long-term dependencies and dynamic properties within the time-series data. When combined with the cross-attention mechanism, the model effectively emphasizes key patterns and their interdependencies, ensuring that relevant information is retained during the learning process and thereby improving overall performance.

In the fault classification phase, the extracted features are mapped and undergo dimensionality reduction through the fully connected layer, which then transforms them into probability distributions for multi-class classification via the softmax layer. The fully connected layer further processes the extracted features, while the softmax layer performs the final classification prediction. In summary, the VFQB model enables accurate bearing fault diagnosis by integrating time-domain and frequency-domain features, dynamic feature extraction, and efficient classification.

## 3. Experiments and Analysis of Results

### 3.1. Introduction to the Datasets

**Figure 4:**
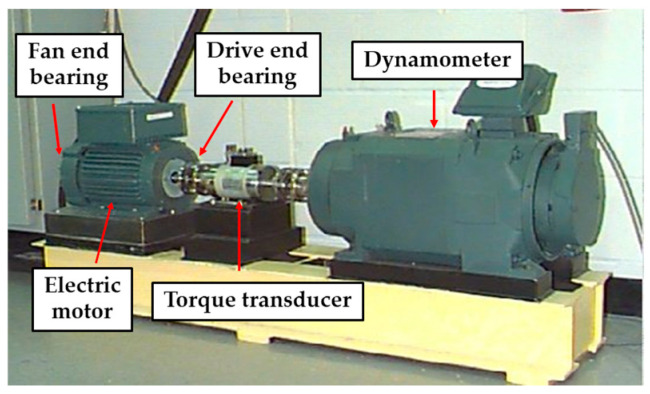
CWRU experimental bench.

The data for Experiment 1 were obtained from the Case Western Reserve University (Cleveland, OH, USA) bearing fault dataset. The experimental setup used for data collection is depicted in Figure 4, which includes a motor, torque transducer, fan end bearing, drive end bearing, and dynamometer. The dataset corresponds to a rolling bearing at the drive end, with a motor speed of 1797 rpm, a load of 0 hp, and a sampling frequency of 12 kHz. The specific details of the data are provided in Table 1. This dataset includes both the healthy operating state and three primary failure scenarios: inner ring failure, outer ring failure, and ball failure. For each failure type, various damage levels were considered: 0.1778 mm, 0.3556 mm, and 0.5334 mm, resulting in a total of 10 distinct failure states for analysis.

**Figure 5:**
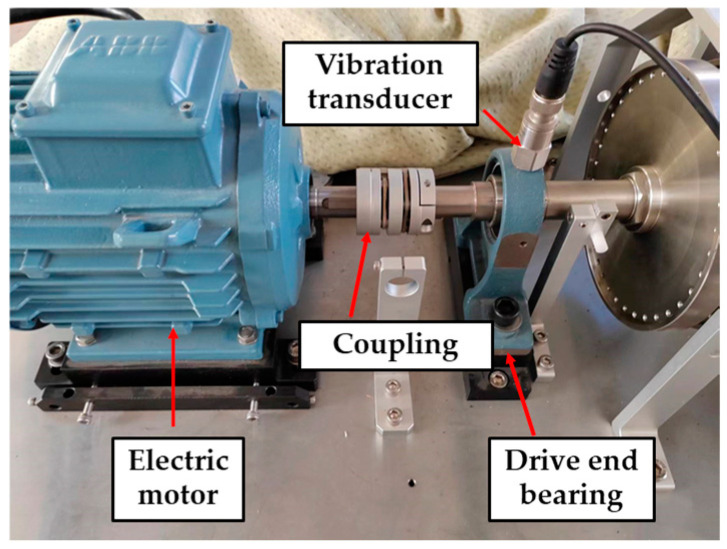
Experiment table of Experiment 2.

The data for Experiment 2 were obtained from laboratory equipment. The experiment table consists of a motor, coupling, drive end bearing, vibration transducer, etc., as shown in Figure 5. This experiment covers six operating conditions: normal operating condition, inner ring failure, outer ring failure, ball failure, cage failure, and combined failure. The operating speed of the bearing is set to 1250 r/min and the load is set to 0 hp; the sampling frequency is 11 kHz, and 16,384 data are collected for each operating condition. The dataset is shown in Table 2.

### 3.2. Model Parameter Setting

**Figure 6:**
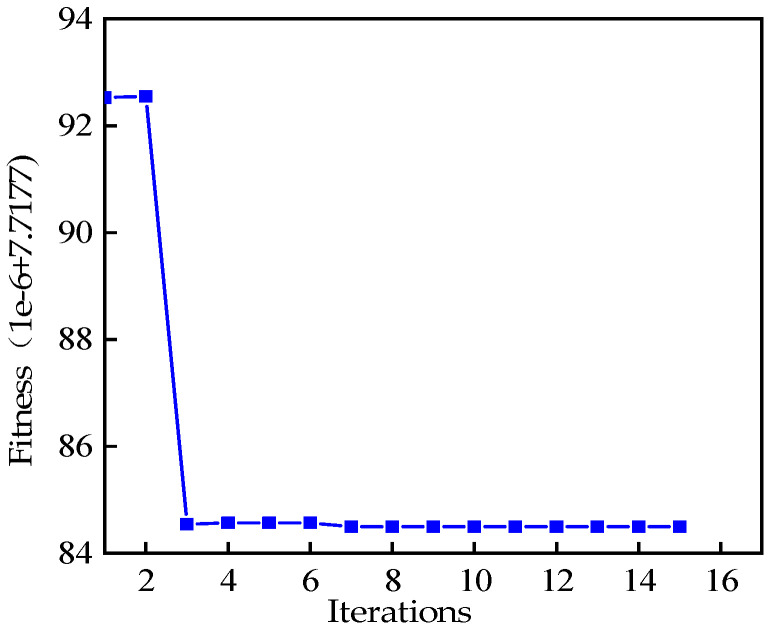
Fitness curve.

The performance of the VMD depends on the selection of key parameters such as the penalty factor α and the modal number *K*. Inappropriate parameters may lead to signal decomposition distortion or modes mix. We introduce the grey wolf optimizer (GWO) algorithm to optimize the VMD parameters to improve the quality of decomposition and the accuracy of signal analysis. The search range of VMD parameters is set $\alpha \in \left[100,2500\right]$, $K\in \left[3,10\right]$; the grey wolf population is set to 20 and the number of iterations is set to 15; the envelope entropy is chosen as the fitness calculation. Figure 6 shows the optimization process of the GWO algorithm with the minimum fitness corresponding to the [*K*, α] combination of [4, 563].

In the quadratic network, the convolution kernel of the convolution layer is set to 1 × 3, a stride of 1, and padding of 1 to ensure that the feature map size remains unchanged after convolution. The kernel size of the max-pooling layer is set to 2, with a stride of 2 and no padding, which reduces the feature map size by half after max pooling. The activation function for both layers is the ReLU function. In the BiGRU network, the activation function is the tanh function. The specific parameters are provided in Table 3.

Two datasets are sampled according to the overlap sampling method with a window of 1024 and an overlap rate of 50%. The sampled data are categorized into training set, validation set, and test set.

### 3.3. Analysis of Experimental Results

**Figure 7:**
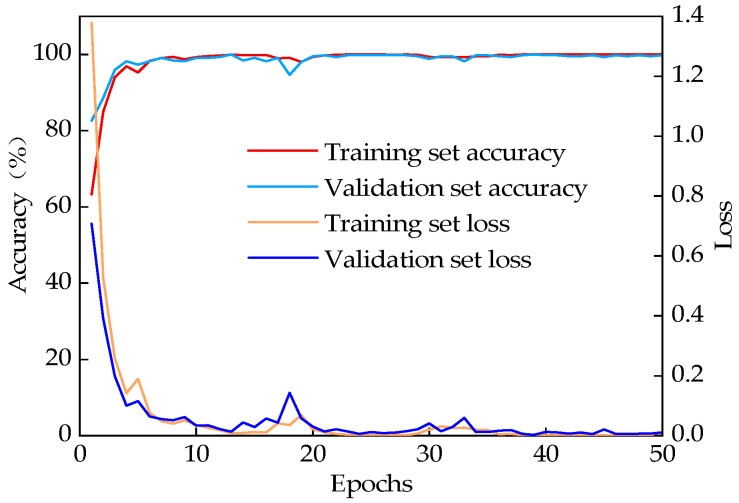
Results of Experiment 1.

**Figure 8:**
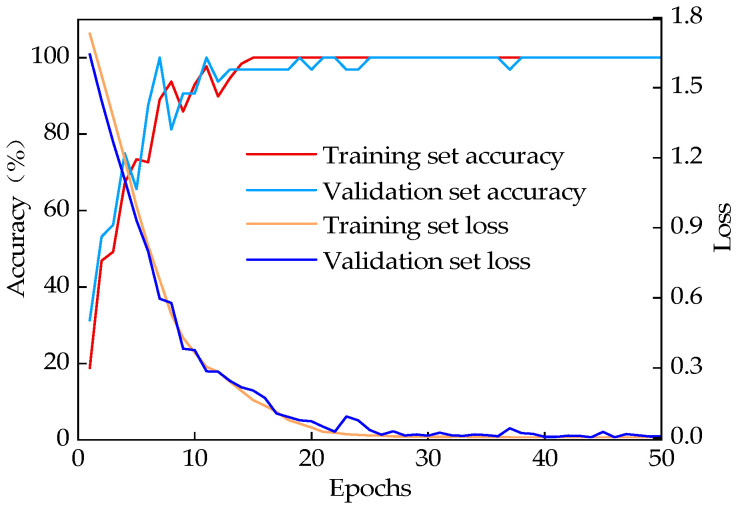
Results of Experiment 2.

The model’s accuracy curve and loss value curves for Experiments 1 and 2 are shown in Figure 7 and Figure 8, following 50 epochs of training. The beginning of training is marked by a low accuracy rate and a high loss value. Increased iterations lead to a gradual increase in accuracy and a stable loss value, indicating that the model is slowly fitting the training data. Furthermore, the high consistency between the training curve and the validation curve suggests that there has been no fitting in the model.

After 20 iterations, the model’s prediction accuracy and loss values stabilized, indicating successful convergence. In Experiment 1, the classification accuracy on the training set reached 100%, with a corresponding loss value of 9.80 × 10^−5^. In Experiment 2, the accuracy also reached 100%, with a loss value of 0.0015. For the validation sets, the classification accuracies were 100% and 100%, with loss values of 0.0001 and 0.007, respectively. These results demonstrate that the combined VFQB model exhibits excellent stability and high accuracy.

**Figure 9:**
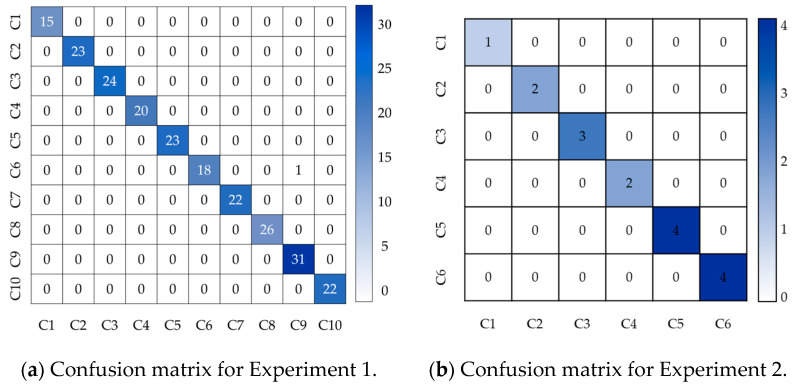
Confusion matrices.

To further visualize the recognition accuracy across different categories in the two experiments, Figure 9 presents the confusion matrix for bearing fault state recognition results. In Figure 9a, the horizontal and vertical axes, labeled C1 to C10, represent the 10 bearing states in Experiment 1, including one normal state and nine fault states. In Figure 9b, the axes labeled C1 to C6 represent the six bearing states in Experiment 2, which include one normal state and five fault states. The diagonal values of each matrix indicate the number of samples in which the model correctly recognized each state.

There is no misidentification of the normal operation state as a fault state in the model, which effectively avoids false shutdowns in actual production. At the same time, when the bearing fails, the VFQB model demonstrates high fault identification accuracy, which can effectively shorten the length of maintenance downtime during the fault and yield substantial economic benefits.

### 3.4. Ablation Experiments

In order to verify the effectiveness of each component of the proposed model, the model was partially disassembled in modules to form three ablation models:

(1) Model 1: This model is constructed by removing the quadratic network module from the original model.
(2) Model 2: This model is constructed by replacing the BiGRU network with a BiLSTM network on the basis of the original model.
(3) Model 3: This model is constructed by removing the attention module from the original model.

The above models are subjected to ablation experiments and evaluated for precision, recall, and F1-score. Table 4 and Table 5 present a comparison of the performance of this paper’s model with the ablation models.

The results show that the diagnostic performance of the proposed model exceeds that of all ablation models. In Experiment 1, the diagnostic accuracy of VFQB is improved by 0.43%, 1.69%, and 0.86% compared to Model 1, Model 2, and Model 3, respectively. In Experiment 2, the accuracy is improved by 2.08% and 4.46% compared to Model 1 and Model 2, respectively, which verifies the contribution of each module to the model’s performance. Specifically, removing the quadratic network module (Model 1) slightly reduces the diagnostic accuracy, indicating that this module contributes to the model’s diagnostic capability to some extent. Replacing the BiGRU with a BiLSTM (Model 2) decreases the diagnostic accuracy compared to the original model, indicating that the BiGRU network is more effective at capturing the dynamic characteristics of the time series data for this task. Removing the attention module (Model 3) has a smaller impact on diagnostic performance, suggesting that the attention mechanism has a relatively limited effect on model enhancement, but still optimizes the attention and extraction of information to some extent. Overall, the original model is able to integrate information more comprehensively by combining various key modules, thereby improving both the accuracy and robustness of the fault diagnosis.

### 3.5. Comparative Experiments

To evaluate the superiority of VFQB in noisy environments, a comparison is conducted with three existing deep learning algorithms: MCNN-LSTM [43], BearingPGA-Net [44], and Laplace_Inception [45]. The experimental results are presented in Table 6 and Table 7.

As observed from Table 6 and Table 7, the diagnostic accuracy of all models decreases to some extent as the signal-to-noise ratio (SNR) decreases. This decline is primarily due to excessive noise, which obscures the characteristics of the original signal. Consequently, as the level of noise increases, the model’s immunity to noise becomes more critical. Among the models evaluated, the VFQB demonstrates superior fault diagnosis accuracy in the −12 to 12 dB SNR range. This can be attributed to the model’s robust feature extraction capabilities, which allow it to effectively identify fault features within the data, even in the presence of noise interference across both the temporal and spatial domains.

When the SNR exceeds 6 dB, all algorithms demonstrate improved fault diagnosis performance in both experiments, achieving accuracy rates above 90% due to the lower noise levels. However, as noise intensity increases, the diagnostic performance of the MCNN-LSTM and Laplace_Inception algorithms deteriorates significantly. Specifically, at an SNR of −12 dB, the accuracy of the MCNN-LSTM model drops to approximately 20%, whereas the VFQB model maintains an accuracy of nearly 85% at the same SNR. This performance can be attributed to the attention module’s ability to focus on important features despite the presence of noise, coupled with the advantages of the BiGRU and quadratic neural network in processing temporal signals. In highly noisy environments with SNRs below 0 dB, the diagnostic accuracy of VFQB decreases to a lesser extent and outperforms the other models. For instance, at −12 dB, the diagnostic accuracy of VFQB in Experiment 1 is 91.17%, which is 69.22% higher than that of MCNN-LSTM, 30.2% higher than BearingPGA-Net, and 9.29 higher than Laplace_Inception. In Experiment 2, the diagnostic accuracy of VFQB is 95.31%, which is 69.02% higher than MCNN-LSTM, 8.21% higher than BearingPGA-Net, and 8.21% higher than Laplace_Inception, highlighting the superior performance of VFQB in high-noise environments. In summary, VFQB is demonstrated through comparative experiments to possess exceptional noise suppression capabilities, effective fault information extraction from vibration signals, and significant potential for intermediary bearing fault diagnosis in noisy environments.

## 4. Conclusions

We proposed a VMD/FFT-Quadratic-BiGRU rolling bearing fault diagnosis model to address the challenges of insufficient feature extraction in traditional fault diagnosis methods and low accuracy under noise interference. The effectiveness of the BiGRU network and the quadratic neural network in extracting signal features is fully verified through comparison and ablation experiments. Through comparisons and analysis with other models in noisy environments, the model proposed in this paper performs well in the evaluation indexes of precision, F1-score, and recall, achieving an accuracy of more than 90%. These results fully demonstrate the model’s superior noise resistance performance and its ability to accurately learn and extract fault information from vibration signals.

## Figures and Tables

**Table 1:** Dataset from Experiment 1.

Serial Number	Fault Type	Failure Size (mm)
0	Normalcy	0
1	Inner ring failure	0.1778
2	Ball failure	0.1778
3	Outer ring failure	0.1778
4	Inner ring failure	0.3556
5	Ball failure	0.3556
6	Outer ring failure	0.3556
7	Inner ring failure	0.5334
8	Ball failure	0.5334
9	Outer ring failure	0.5334

**Table 2:** Dataset from Experiment 2.

Serial Number	Fault Type	Number of Sample Points
0	Normalcy	16,384
1	Inner ring failure	16,384
2	Outer ring failure	16,384
3	Ball failure	16,384
4	Cage failure	16,384
5	Integrated faults	16,384

**Table 3:** Network structure parameters.

Serial Number	Network Layer	Convolution Kernel Sizes	Pacemaker	Padding	Activation Function	Convolution Kernel Quantities	
						Experiment 1	Experiment 2
0	Conv1	1 × 3	1	1	ReLU	32	16
1	Conv2	1 × 3	1	1	ReLU	32	16
2	MaxPool	1 × 2	2	0	-	32	16
3	BN				-	32	16
4	Conv2	1 × 3	1	1	ReLU	64	32
5	Conv4	1 × 3	1	1	ReLU	64	32
6	MaxPool	1 × 2	2	0	-	64	32
7	BN	-	-	-	-	64	32
8	Conv5	1 × 3	1	1	ReLU	128	64
9	Conv6	1 × 3	1	1	ReLU	128	64
10	MaxPool	1 × 2	2	0	-	128	64
11	BN	-	-	-	-	128	64
12	BiGRU	-	-	-	tanh	-	
13	Attention	-	-	-	-	-	
14	Dense	-	-	-	-	-	

**Table 4:** Results of ablation experiments for Experiment 1.

Model	Precision	Recall	F1-Score
Model1	99.57%	99.55%	99.55%
Model2	98.31%	98.21%	98.19%
Model3	99.14%	99.11%	99.11%
VFQB	100%	100%	100%

**Table 5:** Results of ablation experiments for Experiment 2.

Model	Precision	Recall	F1-Score
Model1	97.92%	96.88%	97.08%
Model2	95.54%	93.75%	93.49%
Model3	100.00%	97.50%	92.50%
VFQB	100%	100%	100%

**Table 6:** Comparative experimental results of Experiment 1.

	Model	SNR				
		−12 dB	−6 dB	0 dB	6 dB	12 dB
Precision	MCNN-LSTM	21.95%	38.77%	69.62%	74.05%	88.07%
	BearingPGA-Net	60.97%	91.77%	97.58%	98.84%	99.08%
	Laplace_Inception	82.71%	87.77%	96.41%	98.33%	99.13%
	VFQB	**91.17%**	**95.73%**	**99.14%**	**99.57%**	**100.00%**
F1-score	MCNN-LSTM	20.58%	36.34%	68.19%	73.13%	84.21%
	BearingPGA-Net	56.77%	90.39%	96.35%	98.62%	98.88%
	Laplace_Inception	81.88%	86.50%	95.99%	97.83%	99.11%
	VFQB	**90.73%**	**95.57%**	**99.10%**	**99.55%**	**100.00%**
Recall	MCNN-LSTM	21.37%	38.18%	68.38%	74.36%	84.62%
	BearingPGA-Net	59.17%	90.86%	96.72%	98.64%	98.90%
	Laplace_Inception	81.70%	86.61%	95.98%	97.77%	99.11%
	VFQB	**90.63%**	**95.54%**	**99.11%**	**99.55%**	**100.00%**

**Table 7:** Comparative experimental results of Experiment 2.

	Model	SNR				
		−12 dB	−6 dB	0 dB	6 dB	12 dB
Precision	MCNN-LSTM	26.29%	44.64%	67.24%	80.30%	85.34%
	BearingPGA-Net	87.10%	87.64%	93.99%	96.89%	97.12%
	Laplace_Inception	80.39%	83.97%	89.96%	90.08%	93.18%
	VFQB	**95.31%**	**100.00%**	**100.00%**	**100.00%**	**100.00%**
F1-score	MCNN-LSTM	18.51%	33.43%	65.28%	66.42%	82.89%
	BearingPGA-Net	86.79%	87.33%	94.01%	96.84%	96.84%
	Laplace_Inception	78.68%	63.10%	87.69%	77.52%	90.06%
	VFQB	**93.15%**	**100.00%**	**100.00%**	**100.00%**	**100.00%**
Recall	MCNN-LSTM	17.24%	34.48%	65.52%	68.97%	82.76%
	BearingPGA-Net	87.10%	87.64%	93.99%	96.89%	97.12%
	Laplace_Inception	61.25%	68.75%	87.50%	88.12%	90.62%
	VFQB	**93.75%**	**100.00%**	**100.00%**	**100.00%**	**100.00%**

## Data Availability

Due to privacy issues, we are unable to provide data.

## References

[1] Hu X., Tang T., Tan L., Zhang H. (2023). Fault detection for point machines: A review, challenges, and perspectives. Actuators.

[2] Liu X., Tan J., Long S. (2024). Multi-axis fatigue load spectrum editing for automotive components using generalized S-transform. Int. J. Fatigue.

[3] Wang C., Song Z., Fan H. (2024). Novel evidence theory-based reliability analysis of functionally graded plate considering thermal stress behavior. Aerosp. Sci. Technol..

[4] Liu Z., Liang J., He Z., Liu X., Liu H., Shao Z. (2024). A developed fatigue analysis approach for composite wind turbine blade adhesive joints using finite-element submodeling technique. Eng. Fail. Anal..

[5] Xu M., Han Y., Sun X., Shao Y., Gu F., Ball A. (2022). Vibration characteristics and condition monitoring of internal radial clearance within a ball bearing in a gear-shaft-bearing system. Mech. Syst. Signal Process.

[6] Han Q., Ding Z., Qin Z., Wang T., Xu X., Chu F. (2020). A triboelectric rolling ball bearing with self-powering and self-sensing capabilities. Nano Energy.

[7] Shi J., Zhao B., Niu X., Xin Q., Xu H., Lu X. (2024). Time-varying dynamic characteristic analysis of journal–thrust coupled bearings based on the transient lubrication considering thermal-pressure coupled effect. Phys. Fluids.

[8] Hua L., Liu Y., Qian D., Xie L., Wang F., Wu M. (2022). Mechanism of void healing in cold rolled aeroengine M50 bearing steel under electroshocking treatment: A combined experimental and simulation study. Mater. Charact..

[9] He W., Hang J., Ding S., Sun L., Hua W. (2024). Robust diagnosis of partial demagnetization fault in PMSMs using radial air-gap flux density under complex working conditions. IEEE Trans. Ind. Electron..

[10] Li M., Liu Y., Wang T., Chu F., Peng Z. (2023). Adaptive synchronous demodulation transform with application to analyzing multicomponent signals for machinery fault diagnostics. Mech. Syst. Signal Process.

[11] Zhao D., Cui L., Liu D. (2023). Bearing weak fault feature extraction under time-varying speed conditions based on frequency matching demodulation transform. IEEE/ASME Trans. Mechatron..

[12] Kong Y., Wang T., Chu F. (2019). Meshing frequency modulation assisted empirical wavelet transform for fault diagnosis of wind turbine planetary ring gear. Renew. Energy.

[13] Zhang K., Ma C., Xu Y., Chen P., Du J. (2021). Feature extraction method based on adaptive and concise empirical wavelet transform and its applications in bearing fault diagnosis. Measurement.

[14] Li X., Xie L., Deng B., Lu H., Zhu Y., Yin M., Yin G., Gao W. (2024). Deep dynamic high-order graph convolutional network for wear fault diagnosis of hydrodynamic mechanical seal. Reliab. Eng. Syst. Saf..

[15] Shi J., Zhao B., He J., Lu X. (2024). The optimization design for the journal-thrust couple bearing surface texture based on particle swarm algorithm. Tribol. Int..

[16] Fang X., Zheng J., Jiang B. (2024). A rolling bearing fault diagnosis method based on vibro-acoustic data fusion and fast Fourier transform (FFT). Int. J. Data Sci. Anal..

[17] Wang T., Liang M., Li J., Cheng W. (2014). Rolling element bearing fault diagnosis via fault characteristic order (FCO) analysis. Mech. Syst. Signal Process.

[18] Zhou J., Xiao M., Niu Y., Ji G. (2022). Rolling bearing fault diagnosis based on WGWOA-VMD-SVM. Sensors.

[19] Fan H., Wang C., Li S. (2024). Novel method for reliability optimization design based on rough set theory and hybrid surrogate model. Comput. Methods Appl. Mech. Eng..

[20] Wang S., Zheng C., Ma T., Wang T., Gao S., Dai Q., Han Q., Chu F. (2024). Tooth backlash inspired comb-shaped single-electrode triboelectric nanogenerator for self-powered condition monitoring of gear transmission. Nano Energy.

[21] Hu X., Tan L., Tang T. (2024). M2BIST-SPNet: RUL prediction for railway signaling electromechanical devices. J. Supercomput..

[22] Li B., Tang B., Deng L., Wei J. (2022). Joint attention feature transfer network for gearbox fault diagnosis with imbalanced data. Mech. Syst. Signal Process.

[23] Masalimov K., Muslimov T., Munasypov R. (2022). Real-time monitoring of parameters and diagnostics of the technical condition of small unmanned aerial vehicle’s (UAV) units based on deep BiGRU-CNN models. Drones.

[24] Niu D., Yu M., Sun L., Gao T., Wang K. (2022). Short-term multi-energy load forecasting for integrated energy systems based on CNN-BiGRU optimized by attention mechanism. Appl. Energy.

[25] Zhao Y., Hao H., Chen Y., Zhang Y. (2023). Novelty detection and fault diagnosis method for bearing faults based on the hybrid deep autoencoder network. Electronics.

[26] Li S., Peng Y., Shen Y., Zhao S., Shao H., Bin G., Guo Y., Yang X., Fan C. (2024). Rolling bearing fault diagnosis under data imbalance and variable speed based on adaptive clustering weighted oversampling. Reliab. Eng. Syst. Saf..

[27] Pham M., Kim J., Kim C. (2022). Rolling bearing fault diagnosis based on improved GAN and 2-D representation of acoustic emission signals. IEEE Access.

[28] Li T., Shi H., Bai X., Zhang K. (2023). A digital twin model of life-cycle rolling bearing with multiscale fault evolution combined with different scale local fault extension mechanism. IEEE Trans. Instrum. Meas..

[29] Li K., Wang C., Wu H. (2023). Multimodal transformer for bearing fault diagnosis: A new method based on frequency-time feature decomposition. Res. Sq..

[30] Zhong H., Yu S., Trinh H., Yuan R., Lv Y., Wang Y. (2025). A time-saving fault diagnosis using simplified fast GAN and triple-type data transfer learning. Struct. Health Monit..

[31] Niu M., Ma S., Zhu H., Xu K. (2025). Fault diagnosis of rotating machinery using a signal processing technique and lightweight model based on mechanical structural characteristics. Measurement.

[32] Ding X., He Q., Shao Y., Huang W. (2019). Transient feature extraction based on time–frequency manifold image synthesis for machinery fault diagnosis. IEEE Trans. Instrum. Meas..

[33] Dragomiretskiy K., Zosso D. (2014). Variational Mode Decomposition. IEEE Trans. Signal Process.

[34] Qin C., Huang G., Yu H., Zhang Z., Tao J., Liu C. (2024). Adaptive VMD and multi-stage stabilized transformer-based long-distance forecasting for multiple shield machine tunneling parameters. Autom. Constr..

[35] Cho K., Van Merriënboer B., Gulcehre C., Bahdanau D., Bougares F., Schwenk H., Bengio Y. (2014). Learning phrase representations using RNN encoder-decoder for statistical machine translation. arXiv.

[36] Bao K., Bi J., Ma R., Sun Y., Zhang W., Wang Y. (2023). A spatial-reduction attention-based BiGRU network for water level prediction. Water.

[37] Fantini D., Silva R., Siqueira M., Pinto M., Guimarães M., Brasil A. (2024). Wind speed short-term prediction using recurrent neural network GRU model and stationary wavelet transform GRU hybrid model. Energy Convers. Manag..

[38] Dong Y., Meng Q., Fu J., Ai X., Chen Z., Yin Y. Ultra-short-term load forecasting based on EMD decomposition and CNN-BiGRU heterogeneous computing model. Proceedings of the 2024 IEEE 4th International Conference on Power, Electronics and Computer Applications (ICPECA).

[39] Yang G., Wang X. (2020). Artificial neural networks for neuroscientists: A primer. Neuron.

[40] Fan F., Cong W., Wang G. (2018). A new type of neurons for machine learning. Int. J. Numer. Methods Biomed. Eng..

[41] Fan F., Xiong J., Wang G. (2020). Universal approximation with quadratic deep networks. Neural Netw..

[42] Yu W., Sun J., Zhang S., Zhang X., Liao J. (2023). A class-weighted supervised contrastive learning long-tailed bearing fault diagnosis approach using quadratic neural network. arXiv.

[43] Chen X., Zhang B., Gao D. (2021). Bearing fault diagnosis base on multi-scale CNN and LSTM model. J. Intell. Manuf..

[44] Liao J., Wei S., Xie C., Zeng T., Sun J., Zhang S., Zhang X., Fan F. (2024). BearingPGA-Net: A lightweight and deployable bearing fault diagnosis network via decoupled knowledge distillation and FPGA acceleration. IEEE Trans. Instrum. Meas..

[45] Li T., Zhao Z., Sun C., Cheng L., Chen X., Yan R., Gao R.X. (2022). WaveletKernelNet: An interpretable deep neural network for industrial intelligent diagnosis. IEEE Trans. Syst. Man Cybern. Syst..

